# Pd nanoparticle-modified electrodes for nonenzymatic hydrogen peroxide detection

**DOI:** 10.1186/s11671-015-1021-1

**Published:** 2015-08-04

**Authors:** Jue Wang, Xue-jiao Chen, Kai-ming Liao, Guang-hou Wang, Min Han

**Affiliations:** National Laboratory of Solid State Microstructures and Department of Materials Science and Engineering, Nanjing University, Nanjing, 210093 China; Collaborative Innovation Center of Advanced Microstructures, Nanjing University, Nanjing, 210093 China

**Keywords:** Gas phase cluster beam deposition, Electrocatalytic, Nonenzyme sensors, Hydrogen peroxide detection

## Abstract

A hydrogen peroxide (H_2_O_2_) sensor based on Pd nanoparticles (NPs) and glassy carbon electrodes (GCEs) is fabricated. Pd NPs are deposited on GCEs by using a gas phase cluster beam deposition technique. The NP-deposited electrodes show enhanced electrocatalytic activity in reduction of H_2_O_2_. The electrode with an optimized NP coverage of 85 % has a high selective and stable nonenzymatic sensing ability of H_2_O_2_ with a low detection limit (3.4 × 10^−7^ M), high sensitivity (50.9 μA mM^−1^), and a wide linear range (from 1.0 × 10^−6^ to 6.0 × 10^−3^ M). The reduction peak potential of the electrode is close to −0.12 V, which enables high selective amperometric detection of H_2_O_2_ at a low applied potential.

## Background

Hydrogen peroxide (H_2_O_2_), an important oxidizing, bleaching, and sterilizing agent, is widely used in food processing, pulp and paper bleaching, sterilization, liquid-based fuel cells, and clinical applications [1–7]. It is also an important analyte in food, environmental, and pharmaceutical analyses. Therefore, reliable, accurate, sensitive, rapid, and low-cost analysis of H_2_O_2_ is of great significance. Although several analytical techniques have been employed in the determination of H_2_O_2_, such as titrimetry [8], spectrometry [9], and chemiluminescence [10], most of them exhibit shortcomings like sluggish response, complicated instrumentation, and low sensitivity and selectivity. Electroanalytical method is a preferable alternative [11–14] because of their relatively low cost, better efficiency, ease of operation, high sensitivity, low detection limit, and rapid response. Significant efforts have been expended on designing novel H_2_O_2_ electrochemical sensing techniques and improving their analytical performances.

For the electrochemical sensors, the amperometric determination of H_2_O_2_ requires high overpotential of H_2_O_2_ oxidation and reduction, which limits the selectivity of the sensor. It was found that nanomaterials can reduce the overpotentials for H_2_O_2_ oxidation and reduction and hence minimize the interference from the presence of other chemicals [15, 16]. Metal nanoparticles (NPs), especially those of noble metals, such as platinum [3], silver [17], gold [18], palladium [19], and their hybrids [20], have been commonly employed to perform electrocatalytic H_2_O_2_ detection, due to their fascinating surface-to-volume ratio, favorable biocompatibility, high stability, excellent conductivity, and catalytic ability [21, 22]. Pd NPs are known to have remarkable electrocatalytic activities for various important chemical reactions and have attracted much research interests in the H_2_O_2_ sensor application recently [23]. Sun et al. [24] developed a H_2_O_2_ biosensor, based on immobilization of hemoglobin (Hb) in palladium NPs/graphene-chitosan nanocomposite film. Xiang et al. [25] synthesized onion-like mesoporous carbon vesicle (MCV) with multilayer lamellar structure Pd(25)/MCV/Nafion/GC. Though the enzyme-based biosensors exhibit high sensitivity, they usually suffer from low reproducibility and stability, high cost of enzymes, complicated immobilization procedures, as well as pH value susceptibility. Hence, nonenzymatic H_2_O_2_ sensors based on Pd NPs are also given interest and studied. Pd NPs used for such purpose are commonly synthesized in a solution by a chemical route, such as electrodeposition [26–28], or chemical reductions of suitable Pd-containing species [25, 29, 30]. In such cases, the NPs bind loosely with the electrode and aggregate easily, which results in poor electron transfer and leads to reproducibility and stability problems. Although conducting polymers were widely employed to improve adhesion of the NPs on electrodes, their influence on electrocatalytic activity and response time could not be neglected. Therefore, significant improvement is needed for practical application of metal NP-based devices for H_2_O_2_ detection.

Recently, it was demonstrated that Ag NP-deposited glassy carbon electrode (GCE), fabricated by means of gas phase cluster beam deposition, has the merits of high adhesion, good dispersity, as well as clean surface, owing to the high controllability on the size, coverage, and kinetic energy of the nanoparticles, and shows excellent catalytic activity in the electrochemical reduction of H_2_O_2_ [17]. In this study, we use the gas phase cluster beam deposition process to fabricate Pd NP-deposited GCE (Pd NPs/GCE) and investigate the electrochemical properties of the fabricated electrodes in H_2_O_2_ sensing in detail. We show that by controlling the coverage of Pd NPs, nonenzymatic devices with superior electrocatalytic ability can be fabricated and potentially could be used for commercial application in monitoring the H_2_O_2_ concentration.

## Methods

### Preparation of palladium nanoparticle-deposited GCEs

Pd NPs were produced in gas phase with a magnetron plasma gas aggregation cluster source [31] and deposited on GCEs in a high vacuum chamber. Prior to deposition, GCEs (3 mm diameter) were carefully polished and washed in deionized water to obtain a mirror-like surface. The cluster source was maintained at a gas pressure of 110 Pa by passing 130-sccm argon gas continuously into the aggregation tube. A deposition rate of about 0.4 Å∙s^−1^ was obtained with an input power of 50 W for magnetron discharge. Pd NPs were formed through a gas aggregation process in the argon buffer gas. The nanoparticles were swept out of the aggregation tube along the gas stream to high vacuum chamber through a nozzle and formed a nanoparticle beam with a speed of 1000 m·s^−1^ [32], and got deposited on the GCEs with high adhesion. Four Pd NPs/GCE samples were prepared with different nanoparticle coverage by controlling the deposition time, i.e., for 6, 24, 30, and 40 min. To inspect the morphology and coverage, the deposition was also performed on the transmission electron microscope (TEM) grids, which were attached to the GCEs.

### Reagents

A H_2_O_2_ solution was freshly prepared before use to avoid excessive decomposition. Phosphate buffer saline (PBS; 0.05 M) with pH 7.4 was prepared by Na_2_HPO_4_ and NaH_2_PO_4_ with 0.9 % NaCl as the supporting electrolyte. All reagents were of analytical grade and used as received without any further purification. Double-distilled water was used for the experiment.

### Characterization and electrochemical measurements

The morphology and coverage of the Pd NPs was examined with TEM (TecnaiF20). Electrochemical measurements were carried out on an electrochemical workstation (CHI660D, Shanghai Chenhua Instruments Co., Shanghai, China) by using a three-electrode cell at room temperature. The Pd NPs/GCE, saturated Ag/AgCl electrode, and platinum wire were used as working electrode, reference electrode, and counter electrode, respectively. Cyclic voltammetric and linear sweep stripping voltammetry measurements were performed in PBS at room temperature, which was deaerated with high-purity nitrogen for 10 min. Amperometric measurements at −0.11 V were carried out after successive additions of H_2_O_2_ into PBS in order to provide the convective condition.

## Results and discussion

**Fig. 1:**
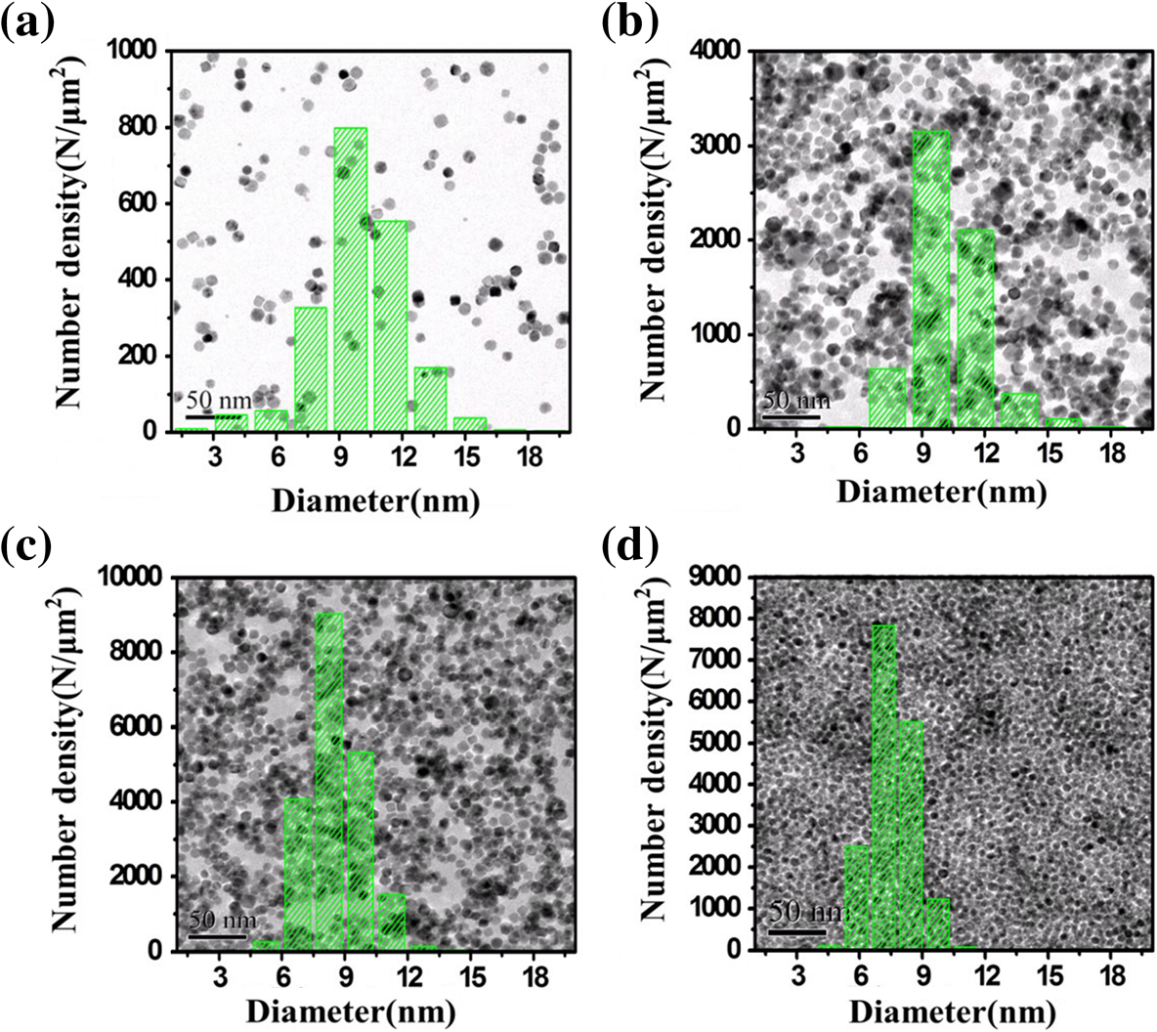
TEM images of the Pd NPs with different nanoparticle coverages (**a** 10, **b** 50, **c** 85, and **d** 115 %) and their corresponding size distributions

TEM images of four films deposited for 6, 24, 30, and 40 min are shown in Fig. 1. The corresponding nanoparticle coverage is measured to be 10, 50, 85, and above 100 %. The mean diameter of the Pd NPs is about 9 nm, which is almost unchanged with the coverage. It clearly indicates that the Pd NPs deposited by a gas phase cluster beam deposition process are comparatively small-sized and cause a nice dispersion, which results in a large specific surface area. Cyclic voltammograms (CVs) and linear sweep stripping voltammetry (LSSV) were obtained for different Pd NPs/GCE in a phosphate buffer solution containing H_2_O_2_.

**Fig. 2:**
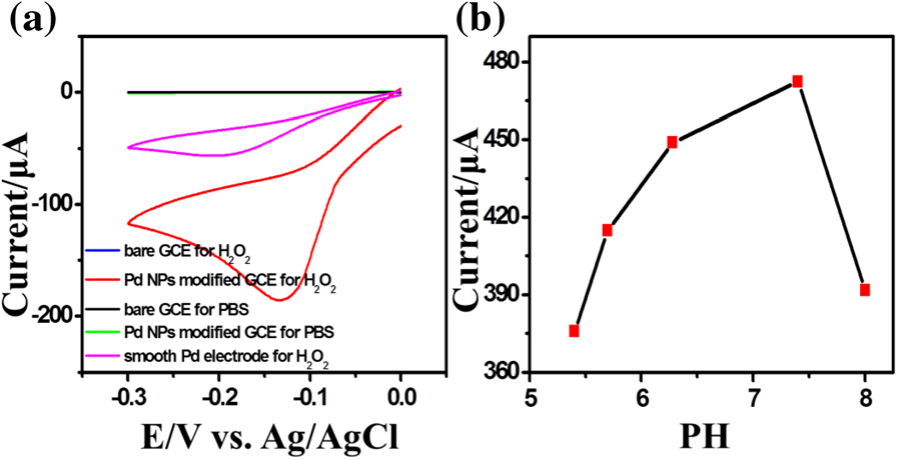
**a** The cyclic voltammograms (CV) of bare and Pd NPs/GCE, smooth Pd electrode in 0.05 M PBS (pH = 7.4) containing 0 M and 0.01 M H_2_O_2_. **b** The effect of solution pH on the current response of the Pd NPs/GCE to 0.01 M H_2_O_2_

Figure 2a shows the CVs obtained for the bare GCE、smooth Pd electrode and Pd NPs/GCE with 10 % coverage in PBS containing 0.01 M H_2_O_2_. As shown for the bare GCE, there is only a very small background current observed in the buffer solution, while for the Pd NPs/GCE, the response current still remains on the background level. Upon addition of 0.01 M H_2_O_2_, no obvious redox response to H_2_O_2_ is observed at the bare GCE over the whole potential range, and smooth Pd electrode shows a weak reduction peak current at −0.2 V. However, for the Pd NPs/GCE, a drastic increase in the reduction current is observed with a well-defined reduction peak at about −0.12 V. It clearly indicates the Pd NPs/GCEs have effective electrocatalytic ability in H_2_O_2_ reduction. The redox peak could be attributed to the enhanced electron transfer between H_2_O_2_ and Pd NPs. The H_2_O_2_ reduction reaction mechanism can be presented by the following equations:

$$ {\mathrm{H}}_2\mathrm{O}{}_2+2{\mathrm{H}}^{+}+2{\mathrm{e}}^{-}\overset{\mathrm{Pd}}{\to }2{\mathrm{H}}_2\mathrm{O} $$

The electrocatalytic activity of the Pd NPs/GCE in reduction of H_2_O_2_ showed a strong dependence on solution pH, as can be seen in Fig. 2b. The reduction peak currents in Fig. 2b were measured from the electrochemical reduction of 0.01 M H_2_O_2_ in the PBS with different pH (5.4 to 8) at a scan rate of 50 mv s^–1^. The current in the reduction of H_2_O_2_ increases with pH and reaches the maximum at pH 7.4 and then drops with further increase in pH. It indicates that the performance of Pd NPs/GCE is optimum at pH 7.4. Therefore, PBS at pH 7.4 was chosen as the electrolyte solution in the voltammetric measurements.

**Fig. 3:**
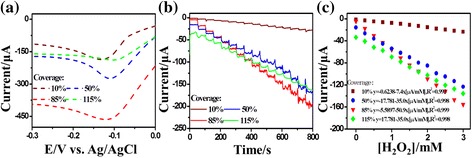
**a** The linear sweep stripping voltammetry (LSSV) curves of the Pd NPs/GCEs with different nanoparticle coverage measured in 0.01 M H_2_O_2_ (0.05 M PBS, pH = 7.4) at the scan rate of 0.05 V s^−1^. **b** The amperometric responses of the Pd NPs/GCEs with different nanoparticle coverage upon successive addition of H_2_O_2_ into gently stirred 0.05 M PBS (pH = 7.4) at −0.11 V. **c** The linear relationship between the response current and the H_2_O_2_ concentration

Previous studies [17, 33] showed that the voltammetric trace for H_2_O_2_ reduction varied with both NP size and the extent of surface coverage. A change in the NP coverage could cause either a change in the reduction peak current or a shift in the peak potential. To optimize the electrode conformation, we performed LSSV measurements on the Pd NPs/GCEs with a different deposition mass and examined the effects of coverage and size distribution of the Pd NPs on the reduction of H_2_O_2_, and the results are shown in Fig. 3a. The morphologies of the nanoparticle arrays can be found in Fig. 1 correspondingly. The LSSV was conducted for 0.01 M H_2_O_2_ in 0.05 M PBS (pH = 7.4) in a potential range from −0.3 V to 0 V (vs. Ag/AgCl) at a scan rate of 50 mv s^−1^ and in saturated N_2_. Since the size of the Pd NPs does not change significantly with the deposition mass, the dependence of LSSV on the deposition mass can be attributed to the difference in the nanoparticle coverage. We can see from Fig. 3a that till nanoparticle coverage approaches a full monolayer, the reduction peak potential as well as the reduction peak current of H_2_O_2_ increase continuously with the coverage. However, when the nanoparticle coverage exceeds one monolayer, the reduction current drops rapidly.

The superior electrocatalytic ability makes the Pd NPs/GCEs promising in the detection of H_2_O_2_. The amperometric *I*-*t* response (instantaneous current *I* vs. time *t*) was measured to detect the H_2_O_2_ reduction reaction at the electrode surface. Figure 3b shows the amperometric *I*-*t* curves of the electrodes with different Pd NP coverage (10–over 100 %). The measurements were performed at the working potential of −0.11 V, recording the instantaneous current change accompanying the stepwise H_2_O_2_ concentration change by successive additions of 25 μL aliquots of 0.1 M H_2_O_2_ in 10 mL of 0.05 M PBS. For each addition, the current increases rapidly with the changes in the H_2_O_2_ concentration and rises to a steady-state current within 10 s, indicating a fast amperometric response to the reduction of H_2_O_2_. Figure 3c shows the corresponding calibration plot of response current against the H_2_O_2_ concentration. It can be found that for every nanoparticle coverage, the response current displays a linear relationship with the concentration of H_2_O_2_ up to at least 3.0 mM, with a sensitivity of 7.4 μA·mM^−1^ for 10 % coverage, 35.0 μA·mM^−1^ for 50 % coverage, 50.9 μA·mM^−1^ for 85 % coverage, and 35.0 μA·mM^−1^ for over 100 % coverage, respectively. It can be seen that for the Pd NPs/GCEs, the sensitivity of H_2_O_2_ detection increases with the NP coverage, except the case in which the coverage is more than 100 %. The increase in sensitivity with the NP coverage is ascribed to the increased nanoparticle density on the electrode surface. The NPs keep well isolated for coverage below one monolayer so that the particle density increases with the coverage. However, when the coverage approaches one monolayer, the Pd NPs start to stack up and coalesce, which reduce the active specific surface area of the nanoparticle arrays and the charge transfer ability between H_2_O_2_ and electrode surface. Therefore, a coverage of 85 % is optimum as it shows the best sensitivity and linearity.

**Fig. 4:**
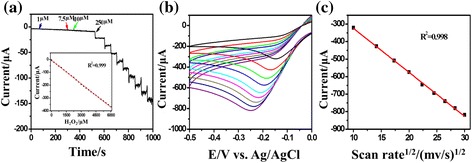
**a** Amperometric response of the Pd NP/GCE (~85 % coverage) upon successive additions of hydrogen peroxide at the potential of −0.11 V. The *inset* shows the corresponding calibration plots. **b** The cyclic voltammograms (CV) of the Pd NP-modified electrode (~85 % coverage) in 0.05 M PBS (pH = 7.4) containing 0.01 M H_2_O_2_ at different scan rates (from top to bottom, 0.1–0.9 V s^−1^). **c** Dependence of the reduction current on the square root of the scan rate

The detection limit and linear response range for H_2_O_2_ detection were evaluated quantitatively for Pd NPs/GCE with 85 % nanoparticle coverage. Fig. 4a shows the amperometric responses of Pd NPs/GCE to the successive additions of H_2_O_2_ at an applied potential of −0.11 V in 0.05 M PBS (pH = 7.4). As shown in the figure, well-defined responses were obtained for the successive additions of 1 μM of H_2_O_2_ at the low H_2_O_2_ concentrations and 250 mM of H_2_O_2_ at the higher H_2_O_2_ concentrations, respectively. The corresponding calibration curve is presented as an inset. The plot displays a linear relationship with the H_2_O_2_ concentration form 1.0 μM to 6.0 mM, with a correlation coefficient of 0.999. The linear response range covers more than three orders of magnitude. The sensitivity determined using the slope of the calibration plot between 1 and 6000 μM was 50.9 μA mM^−1^. A detection limit was determined to be 3.4 × 10^−7^ M, taken as the limiting current for H_2_O_2_ which is three times greater than the standard deviation of the blank. It should be noted that Pd NPs/GCE has a similar sensitivity profile for H_2_O_2_ detection as it was observed in Ag NP-deposited GCE [17]; however, in the present case, linear response range and detection limit are greatly improved. The linear response range is also much better compared to other Pd nanostructure-based nonenzyme electrodes reported recently [19, 26].

**Fig. 5:**
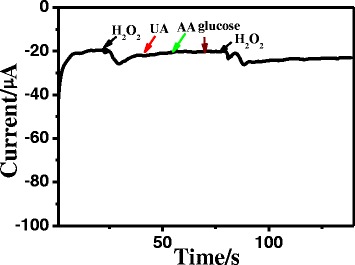
The amperometric response of 0.1 mM hydrogen peroxide, 0.1 mM uric acid, 0.1 mM ascorbic acid, and 0.1 mM glucose at Pd NPs/GCE

Cyclic voltammograms of the Pd NPs/GCE with 85 % nanoparticle coverage were measured at different scan rates from 0.1 to 0.9 V s^−1^. As shown in Fig. 4b, all scan rates result in well-defined reduction peaks between −0.3 and −0.1 V of the electrode potentials, with a little shift in the peak potentials with respect to the scan rate, indicating a kinetic control over the reduction of H_2_O_2_ at the redox sites of Pd NPs/GCE. Furthermore, the reduction peak currents are proportional to the square root of the scan rates (shown in Fig. 4c), which suggests that the electrode reaction is a diffusion control process. It should be noted that the reduction peak potential of the Pd NPs/GCE is close to −0.12 V at a scan rate of 0.1 V s^−1^, which is much smaller than the applied overpotential reported for silver- and palladium-based electrodes [17, 19, 25, 26, 34–36]. To minimize the possible interferences, the overpotentials for H_2_O_2_ oxidation and reduction was tried to reduce [37] in amperometric determination of H_2_O_2_. With an applied potential as small as −0.12 V, almost all the interferences were eliminated and high selectivity to H_2_O_2_ was achieved. We also introduced ascorbic acid, uric acid, and glucose intentionally and studied their effect on the H_2_O_2_ reduction in order to estimate the selectivity to H_2_O_2_. From Fig. 5, we can clearly see with the addition of H_2_O_2_ that the current responds rapidly increased and quickly achieved the steady-state current. The response is not affected by the presence of other chemicals. This particular aspect is appealing. It should be noted that it is a very important requirement for amperometric detection of H_2_O_2_.

It is evident that the Pd NPs/GCEs have high sensitivity, wide linear response range, low detection limit, and high selectivity, indicating that the fabricated device can be potentially used for commercial application in monitoring the H_2_O_2_ concentration. The superior electrocatalytic ability resulted due to the following unique advantages: (a) the small-sized and well-dispersed nanoparticles which provide a high specific surface area, (b) the clean surfaces of the nanoparticles which provide more active sites, and (c) the high density of nanoparticles. Ag NPs are easy to aggregate and coalesce on the carbon surface, so that the size of the NPs increases with the coverage and the specific surface area of the NP arrays is reduced. On the contrary, very high density arrays of Pd NPs are formed on the carbon surface without growth and aggregation. A decrease in NP size sufficiently reduces the overpotentials for H_2_O_2_ oxidation and reduction and minimizes the interference. Furthermore, the Pd NPs not only possess good conductivity but also adhere to the electrode more firmly as they deposit from the cluster beam with relatively high kinetic energy. As a result, easy charge transfer occurs between H_2_O_2_ and the electrode surface and significantly enhances the electrocatalytic activity of the electrodes.

## Conclusions

In summary, we have demonstrated the fabrication of dense Pd nanoparticle arrays covered on the glassy carbon electrode (GCE) and its application for the detection of H_2_O_2_. The nanoparticle arrays were formed by gas phase cluster beam deposition and have the advantages of clean surface, good adhesion ability, and nice dispersion. The Pd NPs/GCEs possess a high specific surface area, allow free access of analytes to the electrode surface, and enable the enhanced electron transfer reaction between H_2_O_2_ and the electrodes. All these features provide a favorable environment for the electrocatalytic reduction of H_2_O_2_ and allow the detection of H_2_O_2_ at a sufficient low applied potential (−0.12 V), which effectively minimizes the interference. We have shown that the electrocatalytic ability of the Pd NPs/GCEs changes with the nanoparticle coverage. The coverage of 85 % is the optimal coverage to achieve both the best sensitivity and linearity. With such optimal nanoparticle coverage, a high selective nonenzyme sensing platform for stable detection of H_2_O_2_ with a low detection limit (3.4 × 10^−7^ M), high sensitivity (50.9 μA mM^−1^), as well as a wide linear range (from 1.0 × 10^−6^ to 6.0 × 10^−3^ M) has been demonstrated. The fabricated device is promising for the development of sensor and biosensor based on nonenzymatic H_2_O_2_ detection.
